# Antibody designing against IIIabc junction (JIIIabc) of HCV IRES through affinity maturation; RNA-Antibody docking and interaction analysis

**DOI:** 10.1371/journal.pone.0291213

**Published:** 2023-09-08

**Authors:** Saima Ejaz, Rehan Zafar Paracha, Sadaf Ejaz, Zunera Jamal

**Affiliations:** 1 School of Interdisciplinary Engineering & Sciences (SINES), National University of Sciences and Technology, Islamabad, Pakistan; 2 Department of Biosciences, COMSATS University Islamabad, Pakistan; 3 Department of Virology, National Institutes of Health, Islamabad, Pakistan; The Islamia University of Bahawalpur Pakistan, PAKISTAN

## Abstract

Hepatitis C virus is a single-stranded RNA based virus which can cause chronic HCV and hepatocellular carcinoma. HCV genotype 3a has relatively higher rate of fibrosis progression, prevalence of steatosis and incidence of HCC. Despite HCVs variation in genomic sequence, the 5’ untranslated region containing internal ribosome entry site (IRES) is highly conserved among all genotypes. It is responsible for translation and initiation of the viral protein. In present study, IRES was targeted by designing variants of reported antigen binding fragment (Fab) through affinity maturation approach. Affinity maturation strategy allowed the rational antibody designing with better biophysical properties and antibody-antigen binding interactions. Complementarity determining regions of reported Fab (wild type) were assessed and docked with IRES. Best generated model of Fab was selected and subjected to alanine scanning Three sets of insilico mutations for variants (V) designing were selected; single (1-71), double (a-j) and triple (I-X). Redocking of IRES-Fab variants consequently enabled the discovery of three variants exhibiting better docking score as compared to the wild type Fab. V1, V39 and V4 exhibited docking scores of -446.51, -446.52 and-446.29 kcal/mol respectively which is better as compared to the wild type Fab that exhibited the docking score of -351.23 kcal/mol. Variants exhibiting better docking score were screened for aggregation propensity by assessing the aggregation prone regions in Fab structure. Total A3D scores of wild type Fab, V1, V4 and V39 were predicted as -315.325, -312.727, -316.967 and -317.545 respectively. It is manifested that solubility of V4 and V39 is comparable to wild type Fab. In future, development and invitro assessment of these promising Fab HCV3 variants is aimed.

## 1 Introduction

Hepatitis C virus (HCV) is a single stranded RNA based virus belonging to the genus Hepacivirus. Globally, around 71 million people suffered from chronic HCV and hepatocellular carcinoma (HCC) with 1.5 million new infections every year [1, 2]. Many patients are unaware of their disease due to the long asymptomatic phase of infection and in long run, 60–70% of chronic infections developed liver cirrhosis and liver cancer. The 9.6 kb long viral genome is bordered by 5’ and 3’ highly structured untranslated regions (UTRs), that hold essential functions such as viral replication, transcription, translation and packaging of the genome into the virions. Both UTRs organize into secondary and tertiary structures that interact with cellular and viral proteins. Besides the 5’ UTR region, the adjacent core genes formed the HCV internal ribosome entry site (IRES). The HCV IRES is responsible for translation & initiation of the viral protein. Moreover, it comprises of 341 nucleotides that fold into four different structural domains. However, the enormous variability observed within individual genotypes of HCV is generated by viral RNA polymerase. The lack of proofreading activity of HCV RNA polymerase accounts for variation and has seven genotypes with 70 subtypes [3, 4].

Despite HCVs variation in genomic sequence, the 5’UTR region containing IRES is highly conserved among all genotypes. The close association between sequence and conservation of IRES determine the effective translation initiation and optimum yield of viral proteins [2]. Several structural and biochemical studies explained the specificity of HCVs IRES and its structural conservation in clinical significance [5, 6]. Therefore, many studies were conducted to explore the correlation between HCVs IRES and patient body response to antiviral therapy. The major obstacle in antiviral treatment is the diversity in HCV IRES viral variants which had a severe impact on virion activity. Among the four domains of HCVs IRES, domain I hold relatively little significance for translational activity while domain II-IV are crucial for translational machinery functioning and are more vulnerable to single-nucleotide substitution or insertion and deletion (indels) mutations [7]. However, domain III is the largest HCV IRES domain, consisting of secondary structural elements such as branched hairpin stem-loops that assembled in 3- and 4-way junctions. The HCV IRES domain III is divided into basal part (IIIef), middle part (IIId) and upper part (IIIabc). While the upper part of HCV contains a 4-way junction (IIIabc) which is essential for the regulation and generation of genomic dimeric isoforms [8]. Moreover, various studies indicate strong interconnections between the formation of dimer and the transitions between viral replication and translation during the viral infectious cycle. Secondary structural elements of HCV IRES serve as a docking site for ribosomal proteins and a hotspot for targeted therapy [9, 10]. Therefore, disruption of the macromolecular interactions i.e., protein-protein interaction between ribosome and domain III of HCV IRES i.e., III abc, demonstrate its implication as a target for small inhibitor molecules as a therapeutic strategy against HCV and associated HCC [11, 12].

High rate of genetic variability in HCV has allow its classification in eight distinct genotypes and more than 50 subtypes. The clinical outcomes of patients infected with HCV vary significantly depending upon the genotype. Among all individuals with HCV infection, HCV genotype 3a infected ones have relatively higher rate of fibrosis progression, incidence of HCC and higher prevalence of steatosis (Grade 3). Despite of direct-acting antiviral (DAA) therapy era, HCV genotype 3a associated infections are relatively problematic to treat as compared to other genotypes of HCV [10]. In spite of rapid development, utilization of inhibitor combinations to target viral entry, resistance in viral strain remains a challenge. However, in previous reports, cell culture models affirmed monoclonal antibodies effectiveness in preventing spread of HCV resistance, but clinical evaluation is still under investigation [13]. Considering the limitations with HCV persistence in infected cell, biotherapeutics promises a solution for HCV eradication from these cells.

It is well established through literature that several approaches developed to inhibit HCV IRES-mediated translation and antibodies serves as a potential therapeutic alternative [5, 9–12]. With continued employment of structural bioinformatics, antibodies have been evolved as versatile binders and recognize surfaces of foreign molecules. Antibodies, the largest class of biotherapeutics is structurally divided in Fc (constant) and Fab (variable) regions. Diverse antibodies are produced by immune system when Fab recognizes and binds with foreign antigens. The binding residues on Fab can be mutated to improve antigen-binding affinity and specificity, a therapeutic approach termed as affinity maturation. If the antigen-antibody interface residues are known, they can be mutated insilico to count on residues that improve the antibody’s binding affinity. Subsequently, paratope-epitope interaction is optimized. The paratope refers to the amino acids on the antibody that come into direct contact with the antigen. The epitope is the term referring to the antigen’s interaction area [13, 14]. To delineate the emerging trends in the therapeutic field and global conformational changes that arise through mutation, computational approaches such as homology modelling, protein interface prediction and protein-protein docking are considered as established standards in antibody discovery [5, 13, 15, 16].

Herein we aim to decode a potential biotherapeutic strategy against HCV by implicating IRES JIIIabc disruption as the functional and drug target motif using the affinity maturation technique. With the advent of affinity maturation strategies, the rational antibody design with better biophysical properties provides the fundamental foundation for the prediction of antibody-antigen binding modes with a tangible impact on biotherapeutic development. In present study, affinity maturation of reported IRES-Fab HCV3 complex PDB ID: 6U8K was accomplished. Moreover, complementarity determining regions (CDRs) of antibody-derived antigen-binding fragments (Fabs) which specifically bind to JIIIabc i.e.,a region exclusively serves as a docking site for 40S ribosome and eukaryotic initiation factor 2 (eIF) [17]. Designing of Fab (Wild type) variants was accomplished that corresponds to better binding affinity to IRES as compared to reported Fab HCV3.

## 2 Methodology

### 2.1 Structure of RNA-Antibody complex

Structure of RNA-antibody complex was retrieved from protein data bank www.rcsb.org, PDB ID: 6U8K [5] with structure resolution of 2.75 Å. Biological assembly 1 comprising of IRES in complex with Fab HCV3 was employed for present study. Corresponding organisms are Hepacivirus C and Homo sapiens respectively. Moreover, expression system employed was Escherichia coli.

### 2.2 Secondary structure annotation of IRES

The significant impact of RNA structure in cellular processes, for instance, gene expression, viral infection and immunity is noteworthy. Structural evaluation of IRES was performed through RNApdbee 2.0 http://rnapdbee.cs.put.poznan.pl/. RNApdbee, an online server performs RNA secondary structure interpretation derived from PBD or mmCIF files [18]. Non canonical base pairing was also assessed because of its critical part in RNA three dimensional structure resulting in RNA functional diversity [19]. Moreover, minimum free energies for optimal and centroid secondary structures were also predicited by using RNAfold 2.4.18 http://rna.tbi.univie.ac.at/cgi-bin/RNAWebSuite/RNAfold.cgi [20].

### 2.3 Assessment of physiochemical properties of IRES and Fab (wild type)

Physiochemical properties of antibody-antigen complex were assessed through ExPASy Protparam tool https://web.expasy.org/protparam/ [21] and DelPhiPKa v2.3 http://compbio.clemson.edu/pka_webserver/ [22]. Physiochemical parameters including the molecular weight, theoretical pI (isoelectric point), amino acids and atomic composition, extinction coefficient, instability & aliphatic index, and grand average of hydropathicity (GRAVY). DelPhiPKa predicts the pKa’s for ionizable groups in proteins, RNA and DNA.

### 2.4 Assessment of CDRs of Fab (wild type)

Complementarity determining regions (CDRs) and framework regions (FRs) are the two types of regions that make up the antibody variable domain. CDRs display a direct connection to antigen-binding, whereas FRs facilitates the CDR framework. Complementarity-determining regions of Fab (Wild type) were assessed through AbRSA tool. Antibody Region-Specific Alignment (AbRSA) http://aligncdr.labshare.cn/aligncdr/index.html is a web service for numbering and CDRs delimiting [23, 24]. Antibody numbering corresponds to IMGT index (international ImMunoGeneTics information system for immunoglobulins) [25].

### 2.5 RNA-Antibody docking and assessment of interface residues

JIIIabc of HCV IRES was docked with Fab (Wild type) by employing HDOCK http://hdock.phys.hust.edu.cn/. Protein docking with protein/DNA/RNA can be performed through HDOCK, developed on a hybrid algorithm of template-based modeling and ab initio free docking [26, 27]. Receptor (IRES) and ligand (Fab (Wild type)) interface residues along with the residue pair(s) at the interface were assessed through HDOCK.

### 2.6 Alanine scanning and insilico mutagenesis

A site-directed mutagenesis approach used to assess the accountability of a specific residue to the given protein in its stability or function is termed as alanine scanning [28]. mCSM-NA https://biosig.lab.uq.edu.au/mcsm_na/ (mutational cutoff scanning matrices) [29] was employed for alanine scanning and single mutations. While mmCSM-NA https://biosig.lab.uq.edu.au/mmcsm_na/ [30] was utilized for ΔΔG prediction in case of multiple mutations. mCSM-NA and mmCSM-NA are based on graph-based signatures in anticipating the effect of mutations on protein–nucleic acid interaction and binding affinities. Chimera 1.10.1 [31] was utilized for performing insilico mutations. Rotamer type is selected with respect to its highest probability from Dunbrack Rotamer library, a backbone-dependent rotamer library comprising the frequencies and standard deviations of the discrete conformations of rotamers [32].

### 2.7 RNA-Antibody redocking & assessment of RNA-Antibody interface

Designed variants predicting increased affinity change or corresponding ΔΔG were selected for redocking. Redocking was accomplished through HDOCK. Moreover, paratope-epitope interface was assessed through interfacial residues pairs [33].

### 2.8 Paratope-Epitope interaction analysis

Paratope-Epitope interactions were calculated through Arpeggio https://biosig.lab.uq.edu.au/arpeggioweb/ [34]. Arpeggio is web tool for evaluation of intra & interatomic interactions in macromolecular structures. Arpeggio is executed in Python and utilizes BioPython [35] and OpenBabel 2.4.1 [36] to read PDB structures. Mutually exclusive, polar and non-polar interactions within 5 Å were calculated between JIIIabc and variants complexes. Model 1 of Fab (Wild type) was utilized as a control. PyMOL 2.5.4 [37] was utilized for visualization of paratope-epitope interactions in complexes and corresponding graphical image generation.

### 2.9 Assessment of aggregation propensity

Protein aggregation is a main obstacle in storage and administration of biotherpeutics. Moreover, aggregation susceptibility results in increased immunogenicity and poor bioavailability of an antibody. Therefore, it necessitates the assessment of aggregation propensity in biotherapeutics structure [38]. Amino acids specific conformation, hydrophobic interactions on protein surfaces and eliminating the imperceptible contribution of hydrophobic residues embedded in the core of folded proteins; all these factors contribute in A3D score of a protein and antibody. Aggregation prone regions (APRs) were assessed through A3D 2.0 server http://biocomp.chem.uw.edu.pl/A3D2/. It aids in the rational design of protein structures and antibodies by predicting the aggregation propensity [38, 39].

## 3 Results

### 3.1 Structural evaluation of IRES

**Fig 1:**
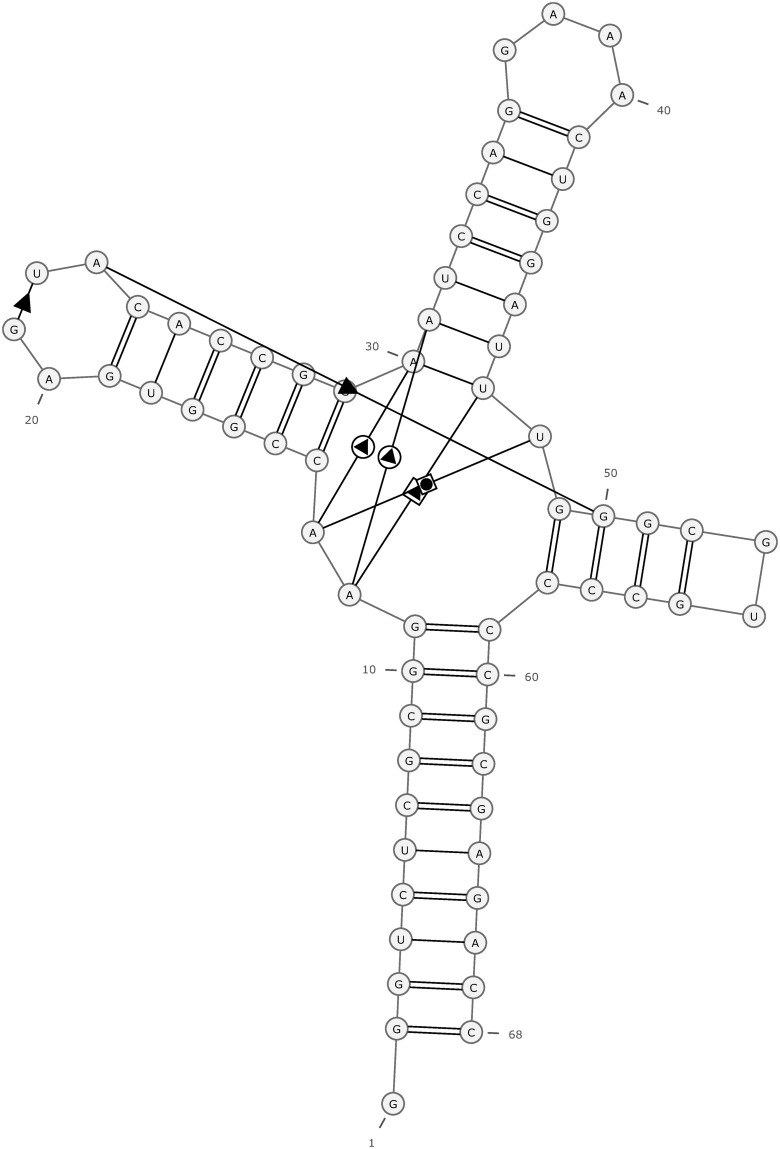
secondary structure orientation of IRES. Fig 1 depicts the secondary structure orientation of IRES extracted from PDB file. Uppercase letters A, C, U and G are RNA base pairs. Labelled numbering 1–68 is showing the length of IRES i.e. 68 Mer. The symbols in figure are depicting the Leontis-Westhof geometric nomenclature and classification of RNA base pairs.

Minimum free energy of RNA optimal and centroid structures was assessed through RNAfold 2.4.18. Moreover, RNApdbee 2.0 was employed for secondary structure prediction and non canonical base pairs. Results were displayed in terms of Base pair, interaction type, Saenger and Leontis-Westhof. Saenger is pattern of nucleic acids base pairing while Leontis-Westhof is geometric nomenclature and classification of RNA base pairs. JIIIabc possesses eight non-canonical base pairs. Detailed results of JIIIabc IRES base pairs with interaction types are presented in Table 1. Moreover, Fig 1 displays the secondary structure annotation of JIIIabc IRES extracted from pdb file.

**Table 1:** IRES non-canonical base pairs with interaction types and corresponding Leontis-Westhof classification.

Non-Canonical Base Pairs in JIIIabc				
S. No	Base-pair	Interaction type	Saenger	Leontis-Westhof
1	A.A154—A.A173	base—base	n/a	W/S cis
2	A.A154—A.U227	base—base	n/a	H/S cis
3	A.A155—A.A172	base—base	n/a	W/S cis
4	A.A155—A.U228	base—base	XXIII	W/S cis
5	A.G163—A.U164	base—base	n/a	S/S cis
6	A.A165—A.G230	base—base	n/a	S/S cis
7	A.U146—A.C247	base—base	n/a	n/a
8	A.A173—A.A225	base—base	n/a	n/a
**Minimum Free Energy of JIIIabc Secondary Structure**				**kcal/mol**
The minimum free energy of optimal secondary structure				41.50 kcal/mol
The minimum free energy of centroid secondary structure				41.50 kcal/mol

Table 1 depicts the IRES non-canonical base pairs with interaction types and corresponding Leontis-Westhof classification. A, U, G and C are RNA bases: Adenine, Uracil, Guanine and Cytosine respectively. n/a stands for not applicable. Saenger is pattern of nucleic acids base pairing while Leontis-Westhof is geometric nomenclature and classification of RNA base pairs [40] XXIII is nucleic acid base pair according to Saenger. W/S cis-Watson-Crick/Sugar Edge with cis bond orientation. H/S cis- Hoogsteen /Sugar Edge with cis bond orientation. S/S cis- Sugar Edge/Sugar Edge with cis bond orientation.

### 3.2 Physiochemical properties of IRES and Fab (wild type)

ExPASy computed physiochemical parameters are displayed in Table 2. Computed instability index (II) of IRES was observed as 61.10 while that of Fab (Wild type) was 52.28. Whereas, aliphatic index of IRES and Fab (Wild type) was 20.59 and 68.01 respectively. GRAVY of IRES was observed as 0.965 while that of Fab (Wild type) was -0.310. Moreover, theoretical isoelectric point (pI) of IRES and Fab (Wild type) was computed as 5.42 and 9.01 respectively. pKa assessment of residues of IRES and Fab (Wild type) were calculated by DelPhiPKa. Detailed results of IRES and Fab (Wild type) pKa are displayed in S1 and S2 Tables respectively.

**Table 2:** Physiochemical parameters of IRES and Fab (Wild type).

Parameter	Fab HCV3	HCV IRES JIIIabc
Molecular weight (Dalton, Da)	46942.33 Da	5945.53 Da
Number of amino acids	437	68
Theoretical pI	9.01	5.42
Extinction coefficient/Abs 0.1% (=1 g/l) assuming all pairs of Cys residues form cystines	69260 M^−1^cm^−1^/1.475	1250
Extinction coefficient/Abs 0.1% (= 1 g/l) assuming all Cys residues are reduced	68760 M^−1^cm^−1^/1.465	0/0.000
Instability index (II)	52.28	61.10
Aliphatic index:	68.01	20.59
Grand average of hydropathicity (GRAVY)	-0.310	0.965
Total number of negatively charged amino acid residues (Asp + Glu)	30	0
Total number of positively charged amino acid residues (Arg + Lys)	40	0

Table 2 depicts the physiochemical parameters of IRES and Fab (Wild type). Extinction coefficients are in units of M^−1^ cm^−1^, at 280 nm measured in water. Abs stands for absorbance. Cys, Asp, Glu, Arg and Lys stand for Cysteine, Aspartic acid, Glutamic acid, Arginine and Lysine resectively.

### 3.3 CDRs assessment and IMGT indexing

**Fig 2:**
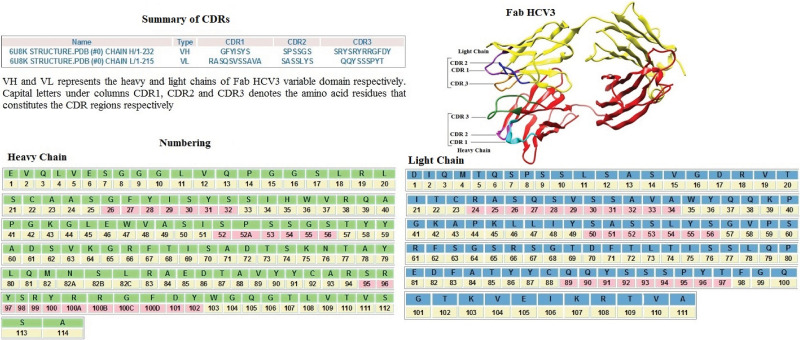
CDRs assessment and IMGT indexing. Fig 2 illustrates the detailed results of AbRSA in terms of Summary of CDRs, Fab (Wild type) and Numbering of Heavy and Light Chains with CDR regions highlighted in pink colour.

AbRSA online tool was employed for CDRs assessment. Fab (Wild type) comprises of heavy and light chain regions. Heavy chain of Fab (Wild type) is composed of the variable and non-variable domains with corresponding residues 1–114 and 215–232 respectively. While, the corresponding residues of variable and non-variable domains for Fab (Wild type) light chain were 1–111 and 112–215 respectively. VH and VL represents the variable domains of heavy and light chain correspondingly. CDR1, CDR2 and CDR3 residues of VH are GFYISYS (26–32), SPSSGS (52–56) and SRYSRYRRGFDY (95–102) correspondingly. Residues of CDRs 1, 2 and 3 regarding VL are RASQSVSSAVA (24–34),SASSLYS (50–56) and QQYSSSPYT (89–97) respectively. Fig 2 depicts the detailed results of AbRSA in terms of CDRs, ribbon diagram of Fab (Wild type) and numbering file of heavy and light chains variable domain.

### 3.4 RNA-Antibody docking & assessment of interface residues

JIIIabc-Fab (Wild type) docking was accomplished through HDOCK. Residues of ligand (Fab (Wild type)) binding site were specified for docking CDR residues 26–32, 52–56, 95–102 of heavy chain (H) and 24–34, 50–56, 89–97 of light chain (L) respectively. Results were displayed with respect to models rank, docking scores, ligand RMSDs and interface residues within 5.0 Å from their interacting chain and the corresponding distances. 100 models were generated. Model 1 was selected for further processing because of its higher binding affinity regarding IRES as compared to the rest of 99 models. Model 1 exhibited a docking score and ligand RMSD of -351.23 kcal/mol and 71.77 Å respectively. Table 3 represents the docking results of first 10 models. Receptor-ligand interface residue pairs within 5Å and the corresponding distances of Model 1 of Fab (Wild type) are enlisted in S3 Table. Interface residue pairing results of IRES- Fab HCV3 (Wild Type) complex showed the participation of CDRs L1, L3, H1, H2 and H3 in interaction. Moreover, pairing of 168A- 67L at a distance of 4.561 in IRES-Fab interaction was also observed in results.

**Table 3:** Docking scores of IRES-Fab (Wild type).

Model Rank	Docking Score	Ligand RMSD
1	-351.23	71.77
2	-323.08	79.13
3	-322.14	93.61
4	-310.88	109.02
5	-306.84	75.69
6	-304.51	79.27
7	-298.48	74.74
8	-296.20	73.80
9	-291.66	67.25
10	-289.41	89.85

Table 3 depicts the Docking scores of IRES-Fab (Wild type) in terms of binding affinity (kcal/mol). RMSD stands for Root Mean Square Deviation of ligand (Fab (Wild type)) measured in Å.

### 3.5 Alanine scanning and prediction of mutations outcome

Results of alanine scanning were displayed in terms of chain, wild type residue with position, mutant, Wild-type RSA% (RSA- relative solvent accessibility) and affinity change. Residues exhibiting decrease in affinity change after being replaced by alanine are selected for insilico mutagenesis. S4 Table depicts the results of alanine scanning. Mutations chosen were based on two criteria: to be (1) embodied within the CDRs; (2) engaged in exhibiting an intermolecular interaction between the antibody (Fab-Wild type) and antigen (IRES). Combinations of mutations have distinct effect on paratope-epitope interaction. Therefore, sets of double and triple mutations were also selected along with single mutations.

Employing the above mentioned criteria, 71 single mutations and set of 10 double and triple mutations each were selected. mCSM-NA and mmCSM-NA were employed for ΔΔG and affinity change prediction in case of single point and multiple point mutations respectively. mmCSM-NA prediction mode was selected that predicts the effects of a set of multiple mutations by considering effects of individual mutations. Results of predictions single mutations were displayed in terms of chain, wild type residue with position, mutant, distance of amino acid residue from interface and affinity change as shown in S5 Table.

### 3.6 Insilico mutagenesis

Out of 71 single mutations and set of 10 double and triple each selected mutation, 39 single mutations with predicted ΔΔG ≥2 were performed using Chimera 1.10.1. However, in case of double and triple mutations no variant (V) with predicted ΔΔG >1 was found. Single mutations are numbered with numerals (1–71) while double and triple mutations are labelled with lower case (a-j) letters and roman numerals (I-X) respectively. S6 Table depicts the insilico mutagenesis results of multiple mutations in terms of variants, chain, wild residue, residue position, mutant residue, distance between the mutated residues and predicted ΔΔG.

### 3.7 Redocking and interface analysis

**Fig 3:**
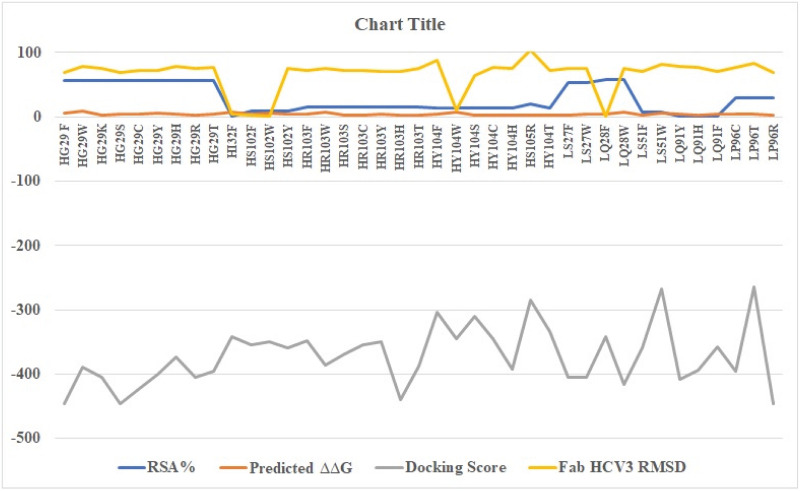
Graphical representation of mutations outcome and docking results of single mutations. Fig 3 depicts the graphical representation of mutations outcome and docking results of single mutations in terms of variants, chain, wild residue, residue position, mutant residue, RSA % (Relative solvent accessibility of the residue), predicted ΔΔG, docking score and ligand RMSD (Root mean square deviation).

**Fig 4:**
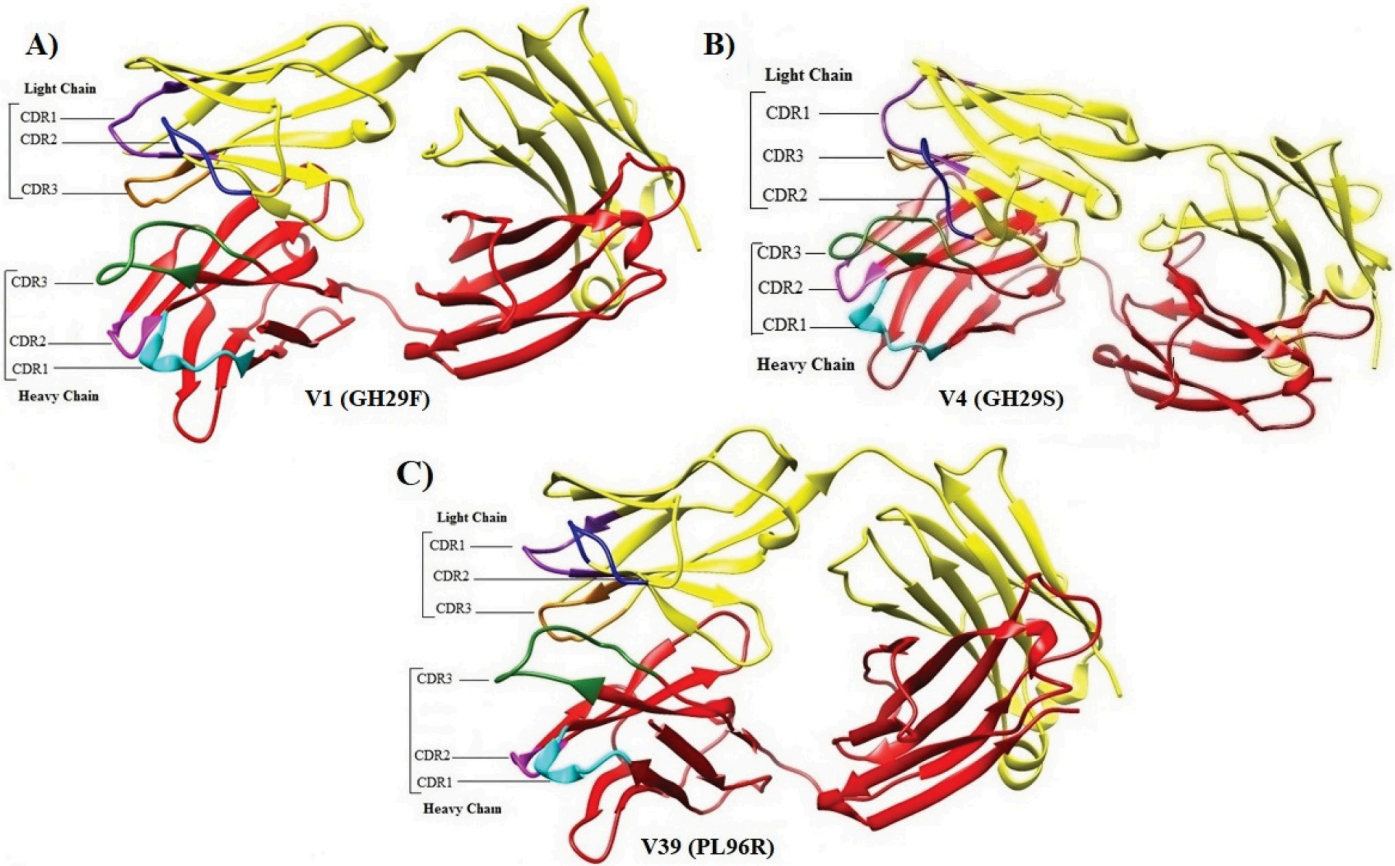
Ribbon models of Variants; V1, V39 and V4. Fig 4 depicts the ribbon diagrams of Variants; V1, V39 and V4 with CDRs of heavy and light chains illustrated. CDR1, CDR2 and CDR3 of heavy chain are labelled in cyan, magenta and forest green colour respectively. While CDR1, CDR2 and CDR3 of light chain are labelled in purple, blue and orange color respectively.

39 single mutations were employed for redocking. Docking was performed through HDOCK. Fig 3 depicts the graphical representation of impact of mutations and docking results of single mutations in terms of variants (V), chain, wild residue, residue position, mutant residue, RSA % (Relative solvent accessibility of the residue), predicted ΔΔG, docking score and ligand RMSD (Root mean square deviation). Corresponding table to the graph is given as S7 Table which shows that out of 39 single mutations V1, V39 and V4 depicted quite good docking score as compared to all of the other variants and Fab (Wild type) as well. V1 (Single mutation- GH29F), V39 (Single mutation- PL96R) and V4 (Single mutation- GH29S) exhibited docking score of -446.51, -446.52 and -446.29 respectively. It seems that mutations performed in designing these variants were favorable in terms of RNA-Antibody interaction. Fig 4 depicts the ribbon diagrams of V1, V39 and V4. Moreover, the receptor-ligand (IRES-Fabs) interface residue pair(s) are given in S8 Table. It enlists the receptor-ligand interface residue pairs within 5Å and the corresponding distances.

### 3.8 Paratope-Epitope interactions

Paratope-Epitope Interactions of Model 1 (Fab-Wild type), V1, V39 and V4 were assessed through Arpeggio. Results were exhibited in terms of mutually exclusive interactions, polar and feature contacts. Mutually exclusive interactions comprises of Van der Waal & Van der Waal Clash interactions, covalent and proximal interactions. Polar contacts include water mediated polar contacts weak polar contacts, and water mediated weak polar contacts. While feature contacts include hydrogen bonds, halogen bonds, aromatic, hydrophobic and carbonyl interactions. Model 1 exhibited 25 and 57 Van der Waal & Van der Waal Clash interactions respectively. In contrast, V1, V4 and V39 exhibited 1497, 1495 and 1497 Van der Waal intercations respectively. In case of polar contacts V1, V39 and V4 showed 55, 54 and 55 interactions respectively. While, Model 1 (Fab-Wild type) exhibited 39 polar contacts only. Likewise, pertaining to hydrogen bonding Model 1 (Fab-Wild type) exhibited 23 hydrogen bonds while V1, V39 and V4 showed 26, 26 and 26 number of hydrogen bonds respectively. Table 4 enlists interface interactions of complexes of designed Fab variants with IRES.

**Table 4:** Interface interactions of designed variants and Fab (Wild Type) with IRES.

Interface Interactions	Fab Wild Type	Variant_1	Variant_4	Variant_39
**Mutually Exclusive Interactions**				
**Total number of contacts**	1292	1497	1495	1497
**Of which VdW interactions**	25	34	39	34
**Of which VdW clash interactions**	57	92	86	92
**Of which covalent interactions**	1	2	2	2
**Of which covalent clash interactions**	0	0	0	0
**Of which proximal**	1209	1369	1368	1369
**Polar Contacts**				
**Polar contacts**	39	55	54	55
**Water mediated polar contacts**	0	0	0	0
**Weak polar contacts**	25	47	45	47
**Water mediated weak polar contacts**	0	0	0	0
**Feature Contacts**				
**Hydrogen bonds**	23	26	26	26
**Water mediated hydrogen bonds**	0	0	0	0
**Weak hydrogen bonds**	7	13	14	13
**Water mediated weak hydrogen bonds**	0	0	0	0
**Halogen bonds**	0	0	0	0
**Ionic interactions**	0	12	12	12
**Metal complex interactions**	0	0	0	0
**Aromatic contacts**	114	156	157	156
**Hydrophobic contacts**	2	3	3	3
**Carbonyl interactions**	6	2	2	2

**Fig 5:**
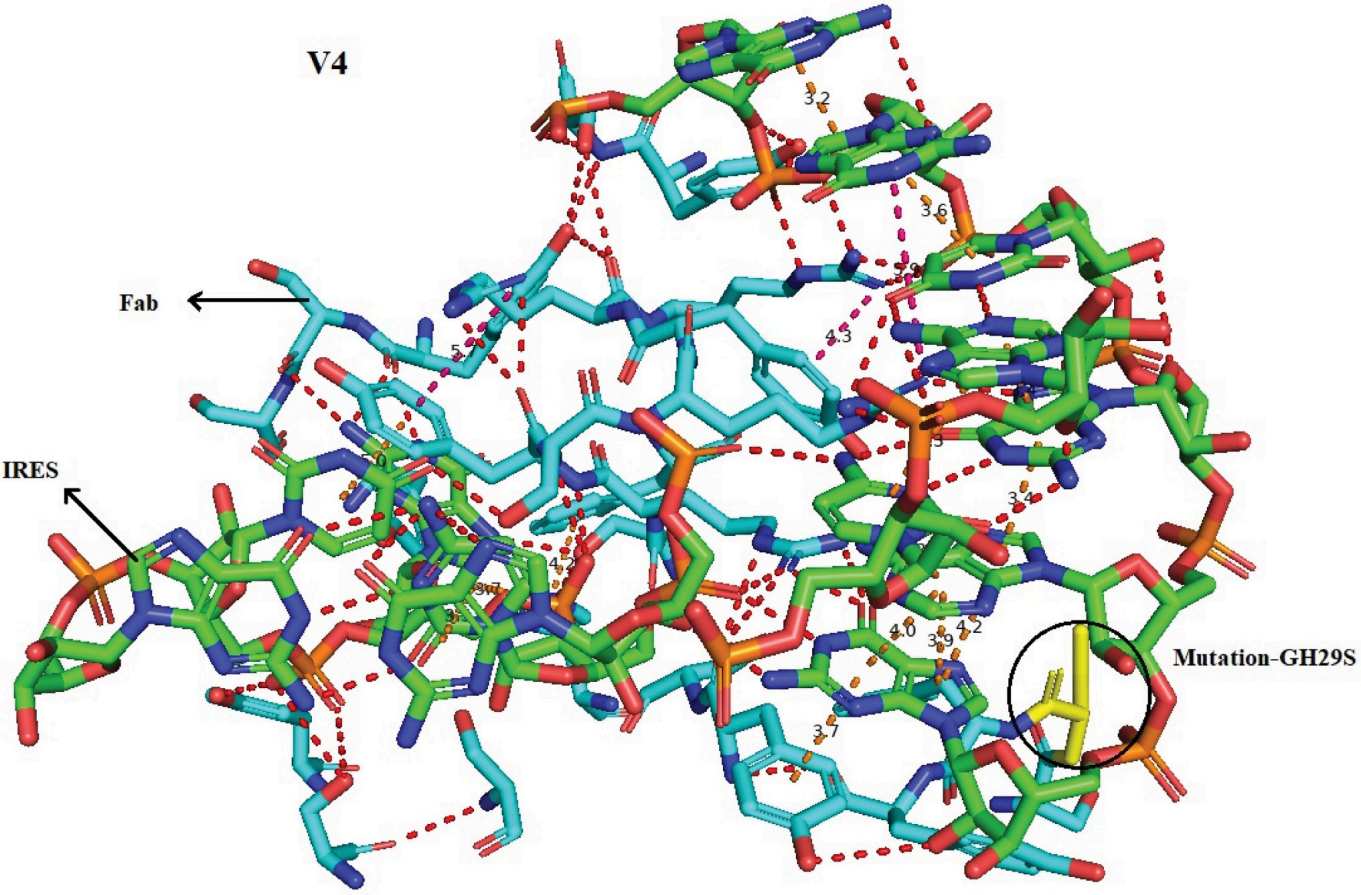
Paratope-epitope interface of IRES-V4 complex. Fig 5 represents the interface residues of V4-IRES complex. Paratope—Epitope interactions are shown as polar contacts, pi-pi and pi-cation intercations in red, orange and hot pink colour dotted lines respectively against light grey background. pi-pi and pi- cation intercations are displayed in dotted lines along with corresponding distances. Green colour chain indicates the IRES residues while cyan colour represents the amino acid residues of Fab region. Moreover, mutated residue is shown in yellow color and labelled in respect to wild type residue, chain, residue number and mutated residue respectively.

Paratope-epitope interface of complexes were visualized in PyMol 2.5.4. Interface residues of complexes were selected and polar contacts, pi-pi and pi-cation intercations were indicated as dotted lines in red, orange and hot pink colour respectively. Green colour chain indicates the IRES residues while cyan colour presents the amino acid residues of Fab region. Moreover, residues of respective chains are also labeled in black colour mentioning the chain IDs as well. Fig 5 depicts the paratope-epitope interface of IRES-V4 complex. Paratope-epitope interfaces of V39 and V1 in complex with IRES are given in S1 Fig.

### 3.9 Aggregation score

**Fig 6:**
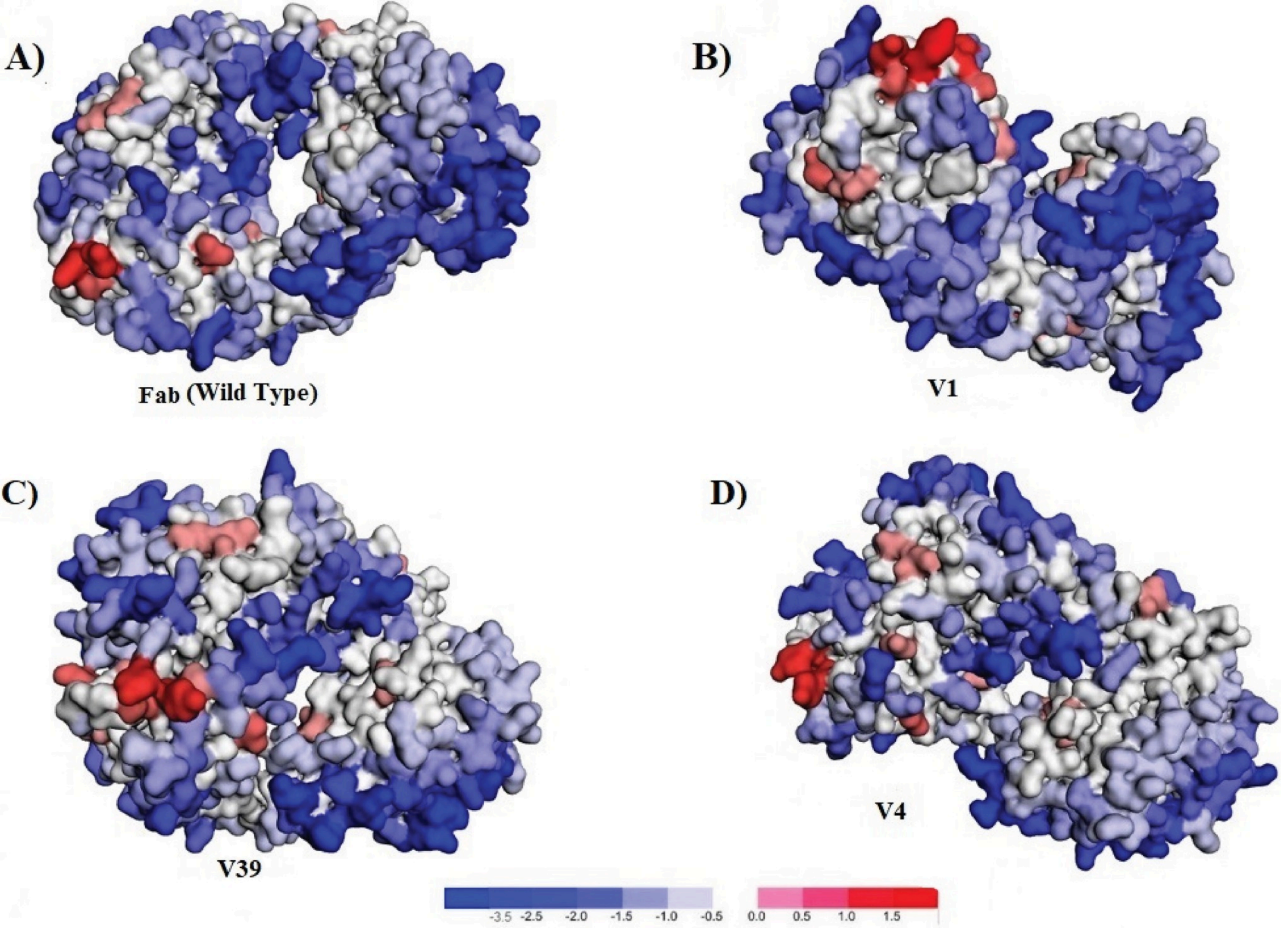
Aggregation prone regions of variants and Fab (Wild type). Fig 6 represents the V1, V4, V39 and Fab (Wild type) displaying the aggregation prone regions. Red colored region indicates aggregation-prone residues while blue colored region represents the soluble residues.

Model 1 (Fab-Wild type), variants V1, V39 and V4 were assessed for their structurally corrected aggregation value (A3D score). Results were given in respect of Minimal and Maximal score of the most soluble and the most aggregation-prone residue in the structural context. Moreover, average and the total A3D score is also predicted. Total A3D scores of Fab (Wild type), V1, V4 and V39 were predicted as -315.325, -312.727, -316.967 and -317.545 respectively as shown in Table 5. Structures of above mentioned variants and Fab (Wild type) displaying the aggregation prone regions are specified in Fig 6. Moreover, S9 Table enlists the A3D Scores of Fab (Wild Type), V1, V4 and V39 in respect to protein, chain ID, residue number, residue name and A3D score respectively.

**Table 5:** Aggregation propensity of Fab (Wild type) and designed variants.

APRs Score	Fab HCV3	Variant 1	Variant 4	Variant 39
Minimal score value (Residue)	-3.9274 (L188E)	-3.5791 (L189K)	-3.9654 (L188E)	-3.8692 (L189K)
Maximal score value (Residue)	1.4014 (H34Y)	1.7897 (H31Y)	1.3606 (H31Y)	1.378 (H31Y)
Average score	-0.7216	-0.7156	-0.7253	-0.7266
Total score value	-315.325	-312.727	-316.967	-317.545

Table 5 depicts the Aggregation Propensity of Fab (Wild type) and Designed Variants. A3D—Structurally corrected aggregation score. APRs—Aggregation Prone Regions. Fab -Fragment antigen binding Region

## 4 Discussion

HCV is a single-stranded RNA-based virus liable for chronic HCV and hepatocellular carcinoma. Its genetic variability is accountable for the variation in clinical outcomes of the patient regarding antiviral therapy. Moreover, its genotype 3a-based infections have a proportionately higher rate of fibrosis, prevalence of steatosis (Grade 3) and the incidence of HCC. Several approaches have been made to inhibit HCV IRES-mediated translation [9, 11, 12]. However, utilization of inhibitor combinations to target viral entry and resistance in viral strains remains a challenge. Although, in previous reports cell culture models affirmed monoclonal antibodies effectiveness in preventing the spread of HCV resistance, nevertheless, the clinical evaluation is still under investigation [13]. Considering the limitations of HCV persistence in infected cell, biotherapeutics promises a solution for HCV eradication from these cells, whereby, antibodies serve as a potential therapeutic alternative [5]. As a response, the current study was formulated that illustrates the designing of Fab (Wild type) variants through an affinity maturation strategy for targeting the IRES region.

Concerningly, the structure (PDB ID: 6U8K) of RNA-antibody complex (IRES—Fab (Wild type)) was downloaded from protein data bank (www.rcsb.org). As per the physiochemical assessment, the predicted instability index (II) of IRES was observed to be 61.10, while that of Fab (Wild type) was 52.28. As the values are above 40, it classifies both the IRES and Fab (Wild type) as unstable [41]. Moreover, the aliphatic index of IRES and Fab (Wild type) is 20.59 and 68.01 respectively, which is regarded as a positive factor that contributes to the thermostability of globular proteins. It is an estimation of aliphatic side chains present in protein [42]. The hydrophobicity of the structure showed the GRAVY of IRES to be 0.965 while that of Fab (Wild type) was -0.310. Generally, hydropathy scale ranges from -2 to +2 for most of the proteins, with a positive value indicating the protein to be more hydrophobic [43]. Therefore, IRES region exhibiting more hydrophobic nature that accounts for structural complexity resulting in its diverse functionality [44].

After docking of reported Fab HCV3 and IRES, 100 models were generated. Model 1 with higher binding affinity and lower RMSD as compared to the rest of the 99 models was selected. Interface residue pairing results of IRES- Fab HCV3 (Wild Type) complex demonstrated the participation of CDRs L1, L3, H1, H2 and H3 in interaction which is in contrast to a reported study [5]. Moreover, the interface study also revealed the pairing of 168A—67L at a distance of 4.561 in IRES-Fab interaction. It might be due to the RNA- Fab docking study that allowed the maximum possible conformations of IRES and Fab.

Model 1 of Wild Type Fab was subjected to insilico alanine scanning. It aids in identifying amino acid residues that are crucial for affinity regarding the differing RNA-antibody binding conformations [28]. Residues exhibiting decreased binding affinity after being substituted by alanine were selected for insilico mutagenesis. Mutations in CDRs of heavy and light chains were performed computationally to anticipate the consequent changes in paratope-epitope interactions. Results showed that out of 71 single mutations and each set of 10 double and triple mutations, 39 single mutations with predicted ΔΔG ≥2 were performed using Chimera 1.10.1. However, in the case of double and triple mutations no variant with predicted ΔΔG >1 was found. Redocking of 39 single mutations demonstrated that V1, V39 and V4 depicted quite a good docking score as compared to all the other variants and Fab (Wild type) as well [26]. V1, V39 and V4 exhibited docking scores of -446.51, -446.52 and -446.29 kcal/mol respectively. It suggests that mutations performed in designing these variants were favorable in terms of IRES-Fab interaction.

An insight into paratope—epitope interface interactions was brought by calculation of interface interactions/contacts. Moreover, the visualization of interface residues with corresponding interactions anticipated that our designed variants-IRES complexes have shown more polar contacts, hydrogen bonds, weak hydrogen bonds and ionic interactions as compared to model 1 (Fab- Wild type). In case of V1 (GH29F), glycine was replaced with phenylalanine which demonstrated an increase in binding affinity. This affinity change is anticipated because of the hydrophobic side chain in phenylalanine in contrast with glycine which constitutes only hydrogen as a side chain group. Hydrophobicity plays an undeniable role in protein folding and unfolding. Moreover, the stabilization of protein-protein interactions extensively depends on the protein hydrophobic nature. However, polar interactions are also engaged in protein interactions [45, 46]. Likewise, in V39 (PL96R), proline was replaced by arginine which constitutes 3 carbon aliphatic chain with a guanidinium group at the distal end. Moreover, in V4 (GH29S) glycine was replaced by serine that possesses a polar uncharged side chain i.e., hydroxymethyl group.Interface analysis of IRES-Fab variants complexes revealed that the mutated residues in V1, V4 and V39 are indirectly involved in interaction with IRES. The side chain of variants poses an impact on overall properties of Fab.

Protein aggregation, an obstacle to biotherapeutic delivery was also addressed by the assessment of aggregation prone motifs in the structure of the designed variant and our control that is Model 1 of Fab (wild type) through A3D score. The average A3D score is a normalized indicator while the total A3D score is a global index of the aggregation tendency or solubility of the protein structure. Effect of constituted amino acid and their substitutions on variation in protein solubility can be anticipated through the A3D score. The more negative the A3D value, the highest the normalized and global solubility. Results demonstrated that variants V4 and V39 have a better aggregation score as compared to Model 1 Fab (wild type). However, V1 displayed an aggregation score of -312.727. Altogether it is manifested that these variants possess comparative solubility features as Model 1 of Fab (wild type).

## 5 Conclusion

A set of three novel Fab HCV3 variants were designed against IRES through an antibody affinity maturation approach. Insilico mutagenesis strategy was employed for designing variants of reported Fab HCV3. Single and multiple mutations were performed for deigning variants against IRES. V1, V39 and V4 exhibited docking scores of -446.51, -446.52 and -446.29 kcal/mol respectively which is better as compared to the Fab (wild type) that exhibited -351.23 kcal/mol. It is manifested that mutations for designing these variants are favorable in terms of IRES-Fab binding interactions. Moreover, the aggregation score of variants and Fab (wild type) were also assessed. Total A3D scores of Fab (Wild type), V1, V4 and V39 were predicted as -315.325, -312.727, -316.967 and -317.545 respectively. It is concluded that the solubility of V1, V4 and V39 is comparable to Fab (wild type). Altogether, it is anticipated that aforementioned three variants V1, V4 and V39 are promising Fabs HCV3. In the future, development and assessment of these Fab HCV3 variants is aimed.

## Supporting information

S1 Table pKa values of HCV IRES JIIIabc residues. (XLSX)

S2 Table pKa values of Fab HCV3 residues. (XLSX)

S3 Table Receptor-ligand interface residue pairs of Model 1 of Fab HCV3 and IRES complex. S3 Table enlists the receptor-ligand interface residue pairs within 5Å and the corresponding distances of Model 1 of Fab HCV3 and IRES complex.
(XLSX)

S4 Table Results of alanine scanning. S4 Table represents the results of alanine scanning in terms of chain, wild type residue with position, mutant, wild-type RSA% (RSA- relative solvent accessibility) and affinity change.
(XLSX)

S5 Table Results of ΔΔG prediction of 71 single mutations. S5 Table represents results of ΔΔG prediction of 71 single mutations displayed in terms of chain, wild type residue with position, mutant, distance of amino acid residue from interface and affinity change.
(XLSX)

S6 Table Results of insilico mutagenesis in case of multiple mutations. S6 Table depicts the insilico mutagenesis results of multiple mutations in terms of variants, chain, wild residue, residue position, mutant residue, distance between the mutated residues, predicted ΔΔG, docking score and ligand RMSD.
(XLSX)

S7 Table ΔΔG prediction. S7 Table represents results of ΔΔG prediction displayed in terms of chain, wild type residue with position, mutant, distance of amino acid residue from interface and affinity change.
(XLSX)

S8 Table Receptor-ligand interface residue pairs of Var 1, Var 4 and Var 39. S8 Table enlists the receptor-ligand (HCV IRES JIIIabc-Fab HCV3) interface residue pairs of Var 1, Var 4 and Var 39 within 5Å and the corresponding distances.
(XLSX)

S9 Table A3D Scores of Fab (wild Type), V1, V4 and V39. S9 Table enlists the A3D scores of Fab (wild Type), V1, V4 and V39 in respect to protein, chain, residue number, residue name and A3D score respectively.
(XLSX)

S1 File (BST)

S2 File (BIB)

S1 Fig Paratope-epitope interface of IRES-V1 & IRES-V39 complex. S1 Fig: A and B represents the interface residues of V1-IRES and V39-IRES complexes respectively. Paratope—Epitope interactions are shown as polar contacts, pi-pi and pi-cation interactions in red, orange and hot pink color dotted lines respectively against light grey background. pi-pi and pi- cation interactions are displayed in dotted lines along with corresponding distances. Green color chain indicates the IRES residues while cyan color represents the amino acid residues of Fab region. Moreover, mutated residue is shown in yellow color and labelled in respect to wild type residue, chain, residue number and mutated residue respectively.
(TIF)
