# Supplementary figures and images for: Antibody designing against IIIabc junction (JIIIabc) of HCV IRES through affinity maturation; RNA-Antibody docking and interaction analysis
Source: PLoS One. 2023 Sep 8;18(9):e0291213. doi: 10.1371/journal.pone.0291213 (PMC10490861; doi:10.1371/journal.pone.0291213)

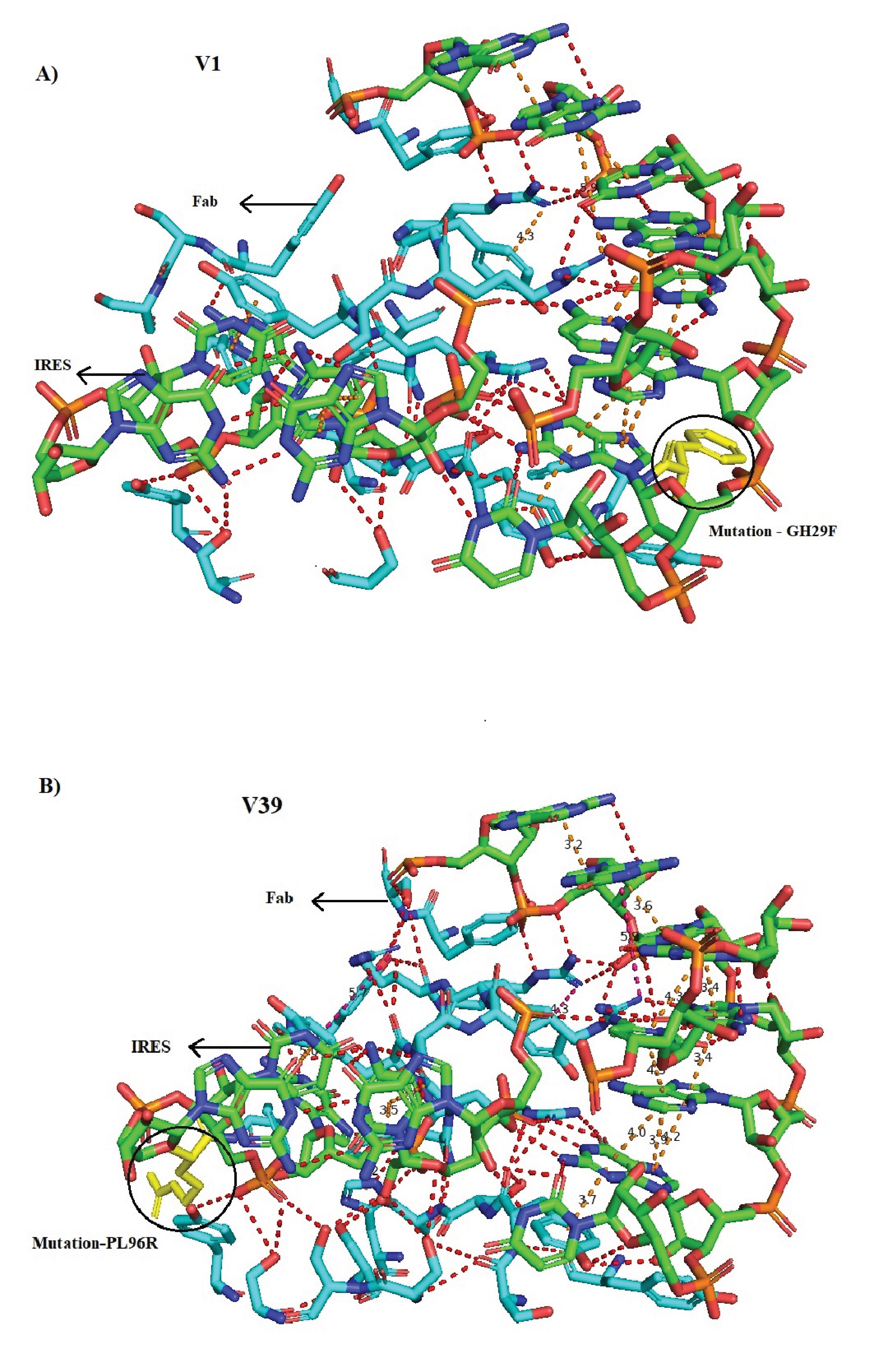

Supplement: S1 Fig — A and B represents the interface residues of V1-IRES and V39-IRES complexes respectively. Paratope—Epitope interactions are shown as polar contacts, pi-pi and pi-cation interactions in red, orange and hot pink color dotted lines respectively against light grey background. pi-pi and pi- cation interactions are displayed in dotted lines along with corresponding distances. Green color chain indicates the IRES residues while cyan color represents the amino acid residues of Fab region. Moreover, mutated residue is shown in yellow color and labelled in respect to wild type residue, chain, residue number and mutated residue respectively. (TIF) [file pone.0291213.s012.tif]
